# Translating peripheral blood biomarkers into Alzheimer’s disease care: an exploratory dual-pathway model of immune and viral dynamics in behavioral and psychological symptoms of dementia

**DOI:** 10.3389/fnagi.2026.1867226

**Published:** 2026-08-05

**Authors:** Ching-Tse Wu, Shuo-Peng Chou, Chuang-Rung Chang, Chien-Chung Chang, Cheng-I Chu

**Affiliations:** 1 Institute of Biotechnology, National Tsing Hua University, Hsinchu, Taiwan; 2 Department of Laboratory Medicine, National Taiwan University Hospital Hsin-Chu Branch, Hsinchu, Taiwan; 3 Institute of Cellular Biology, National Tsing Hua University, Hsinchu, Taiwan; 4 Department of Neurology, National Taiwan University Hospital Hsin-Chu Branch, Hsinchu, Taiwan

**Keywords:** Alzheimer’s disease, BPSD, immunosenescence, neuroinflammation, peripheral biomarkers, virus

## Abstract

**Background:**

Behavioral and psychological symptoms of dementia (BPSD) critically complicate Alzheimer’s disease care, yet the precise biological correlates for highly heterogeneous symptom domains remain largely unexplored.

**Objective:**

To investigate an exploratory “dual-pathway framework” to map how macroscopic immunosenescence and elevated latent viral antibody titers parallelly correlate with distinct neurobehavioral phenotypes.

**Methods:**

We evaluated 103 older adults diagnosed with mild cognitive impairment (n = 40) or probable Alzheimer’s disease (*n* = 63). The severity and frequency of behavioral domains were assessed using the Neuropsychiatric Inventory (NPI). These profiles were correlated with peripheral blood lymphocyte subsets and serological markers of neurotropic Herpesviridae.

**Results:**

Macroscopic immunosenescence, reflected by an elevated CD4/CD8 ratio (>1.58), differentiated Alzheimer’s disease from mild cognitive impairment (*p* = 0.028), indicating cross-sectional clinical status. To address the highly zero-inflated distribution of BPSD symptoms, we employed a two-step hurdle-like approach. Initial comparisons revealed that elevated EBV-VCA IgM titers were significantly associated with the occurrence of BPSD symptoms (*p* = 0.0475). However, subsequent severity analyses exclusively within symptomatic subsets showed that viral titers lacked a continuous linear correlation with symptom severity. Instead, the reduced absolute counts of adaptive immune cells (CD3+, CD4+, and CD19+) exhibited a robust, dose-dependent negative correlation with the global neurobehavioral burden (Total NPI score).

**Conclusion:**

Behavioral and psychological symptoms of dementia are associated with an exploratory dual-pathway model. Elevated viral antibody titers act as an immunological priming factor associated with symptom occurrence, whereas the reduction of adaptive immune reserves continuously modulates the global neuropsychiatric severity in Alzheimer’s disease.

## 1 Introduction

Behavioral and psychological symptoms of dementia (BPSD) represent the most challenging aspect of Alzheimer’s disease (AD) care. While prior studies acknowledge the involvement of peripheral lymphocyte alterations and viral infections in AD pathogenesis, the literature lacks the granularity to delineate how these systemic biological changes map onto specific BPSD domains. Growing evidence indicates that aberrant immune responses and neuroinflammation contribute to neuropsychiatric disturbances, yet the precise immunological triggers for distinct symptoms–such as psychosis or hyperactivity–remain largely unexplored.

The viral hypothesis of AD has gained substantial traction, suggesting that amyloid-beta may initially serve as an antimicrobial defense before driving chronic neuroinflammation (Wainberg et al., 2021). Studies have frequently detected Herpesviridae, particularly the Epstein-Barr virus (EBV), in AD brain tissues and peripheral blood (Allnutt et al., 2020; Carbone et al., 2014). Concurrently, aging and neurodegeneration are characterized by systemic immune senescence, notably a shift in T-cell homeostasis and a decline in B-cell regulatory functions. We suggest that this immunological deterioration compromises the control of latent neurotropic viruses, allowing for their elevated latent viral activity and the subsequent exacerbation of neuroinflammation.

To address this translational gap, we propose an exploratory “dual-pathway model” underlying BPSD. We hypothesize that macroscopic immune senescence–reflected by systemic T-cell imbalances (e.g., an elevated CD4/CD8 ratio)–serves as the immunological background indicating advanced clinical disease stages. Building upon this vulnerable environment, we propose a functional bifurcation in the pathogenesis of BPSD: elevated viral antibody titers (e.g., EBV-VCA IgM) act as an immunological priming factor associated with the occurrence of behavioral symptoms, while the subsequent reduction of adaptive immune reserves (e.g., diminished absolute counts of CD3+, CD4+, and CD19+ cells) serves as a continuous modulator dictating global neurobehavioral severity. By utilizing clinically accessible peripheral biomarkers and assessing twelve BPSD domains via the Neuropsychiatric Inventory (NPI), this study aims to establish precise associations between this conceptual framework and the neurobehavioral burden. Ultimately, this approach seeks to bridge the bench-to-bedside gap, offering a biologically grounded framework to inform future immunomodulatory and antiviral strategies in dementia care.

## 2 Materials and methods

### 2.1 Ethics statement

The study protocol was approved by the Research Ethics Committee of National Taiwan University Hospital (NTUH-REC No.: 202307203RINA). A waiver of informed consent was granted due to the retrospective nature of the chart review and minimal risk to participants. The study was conducted in full adherence to the Declaration of Helsinki.

### 2.2 Participants and clinical diagnosis

A retrospective chart review was conducted for 103 older adults (aged > 65 years) presenting with primary short-term memory impairment between 2022 and 2024. Patients were classified into an MCI group (*n* = 40) and a probable AD group (*n* = 63) based on comprehensive clinical consensus (Table 1). All participants were diagnosed with clinically probable Alzheimer’s disease in strict accordance with the 2011 NIA-AA core clinical criteria (McKhann et al., 2011). While we acknowledge the paradigm shift toward a biological definition of AD in the 2024 NIA-AA revised criteria (Jack et al., 2024), advanced central molecular diagnostics were not routinely employed in our cohort due to real-world clinical and economic constraints in standard practice. Therefore, a rigorous clinical “diagnosis of exclusion” was enforced. Comprehensive biochemical evaluations and structural neuroimaging were systematically utilized to exclude reversible dementias, major cerebrovascular diseases, and brain tumors. Patients with a history of major psychiatric illnesses or severe head trauma were also strictly excluded.

**TABLE 1:** Demographic, clinical, and immunological characteristics of the study cohort.

Characteristic/variable	MCI (*n* = 40)	AD (*n* = 63)	*P*-value
Demographics and cognition			
Age (years), mean ± SD	74.23 ± 8.54	83.05 ± 6.66	<0.001*
Female, *n* (%)	25 (62.5%)	35 (55.5%)	–
MMSE, mean ± SD	24.18 ± 3.94	14.62 ± 6.47	<0.001*
CDR-SOB, mean ± SD	1.65 ± 1.15	6.29 ± 3.90	<0.001*
Immune and viral biomarkers			
CD4/CD8 ratio, mean ± SD	1.87 ± 1.24	2.26 ± 1.12	0.028*
EBV-VCA IgM positive rate (>9), *n* (%)	2 (5%)	2 (3%)	–
EBV-VCA IgM titer, mean ± SD	2.26 ± 2.07	2.72 ± 3.17	0.437
Key neuropsychiatric symptoms (NPI)			
Total NPI score, mean ± SD	6.28 ± 6.00	16.62 ± 15.42	<0.001*
Nighttime behavior, prevalence (%)	45.00%	58.70%	–
Nighttime behavior, score	2.63 ± 3.51	3.89 ± 4.25	0.152
Apathy, prevalence (%)	20.00%	36.50%	–
Apathy, score	0.48 ± 1.36	2.30 ± 3.69	0.023*
Anxiety, prevalence (%)	25.00%	22.20%	–
Anxiety, score	0.30 ± 0.61	0.46 ± 1.12	0.932

Data are presented as mean ± SD unless otherwise specified. Asterisks (*) indicate statistical significance (*p* < 0.05).

### 2.3 Neuropsychological and behavioral assessments

Cognitive staging was evaluated using the Taiwanese version of the Mini-Mental State Examination (MMSE) and the Clinical Dementia Rating Scale Sum of Boxes (CDR-SOB). The Neuropsychiatric Inventory (NPI) was employed to assess the frequency and severity of 12 BPSD domains. Each domain score was calculated as frequency (Allnutt et al., 2020; Carbone et al., 2014; McKhann et al., 2011; Wainberg et al., 2021) multiplied by severity (Allnutt et al., 2020; Carbone et al., 2014; Wainberg et al., 2021), with higher total scores reflecting greater BPSD burden. Assessments were administered by well-trained clinical psychologists.

### 2.4 Peripheral blood biomarkers and viral assays

Fasting peripheral blood samples were collected during routine clinical visits. For the quantification of lymphocyte subsets, peripheral blood mononuclear cells (PBMCs) were isolated and stained with specific fluorochrome-conjugated monoclonal antibodies targeting CD3, CD4, CD8, and CD19 (Beckman Coulter). Flow cytometry acquisition and analysis were performed at the Department of Laboratory Medicine, National Taiwan University Hospital Hsin-Chu Branch, utilizing a Beckman Coulter Navios EX flow cytometer.

Concurrently, specific antibody titers against neurotropic Herpesviridae were measured using two distinct automated clinical platforms. Epstein-Barr virus (EBV-VCA IgG/IgM) and Varicella-Zoster virus (VZV IgG) levels were assessed using commercially available enzyme-linked immunosorbent assay (ELISA) kits processed on a ThunderBolt automated EIA/CLIA analyzer. Herpes simplex virus (HSV 1/2 IgM, HSV 1 IgG, HSV 2 IgG) titers were quantified using chemiluminescent immunoassay (CLIA) kits on a LIAISON XL fully automated analyzer. All assays were executed with quality control and calibration conducted strictly according to the respective manufacturers’ standardized protocols.

### 2.5 Statistical analysis

Statistical analyses were performed using GraphPad Prism. Given the non-parametric distribution of the immunological and behavioral variables (confirmed via Shapiro-Wilk test), the Mann-Whitney U test was utilized to compare variations between groups. Furthermore, Receiver Operating Characteristic (ROC) curve analysis and Youden’s Index were employed to identify the optimal cut-off value of the CD4/CD8 ratio for distinguishing AD clinical disease stages. Two-sided *p*-values < 0.05 were considered statistically significant. Given the exploratory nature of mapping specific subsets to 12 distinct domains, formal adjustments for multiple comparisons were not strictly applied to minimize Type II errors in this novel dual-pathway investigation. To rigorously address the heavy zero-inflation inherent in Neuropsychiatric Inventory (NPI) scores and delineate the distinct roles of viral and immune markers in BPSD, we implemented a modified two-step hurdle-like approach. First, to assess the threshold associated with symptom occurrence (presence vs. absence), Mann-Whitney U tests were utilized to compare peripheral viral and immune metrics between asymptomatic (NPI score = 0) and symptomatic (NPI score > 0) patients. Second, to evaluate continuous symptom severity without the mathematical distortion of tied zero ranks, Spearman rank correlations and simple linear regressions were performed exclusively within the symptomatic subsets. Furthermore, to control for the inflation of Type I errors during multiple correlation analyses between continuous viral/immune parameters and NPI scores, we applied the False Discovery Rate (FDR) correction utilizing the two-stage linear step-up procedure of Benjamini, Krieger, and Yekutieli, with a desired *Q*-value of 5% (*q* = 0.05).

## 3 Results

### 3.1 Patient characteristics and CD4/CD8 ratio as a staging biomarker

**FIGURE 1:**
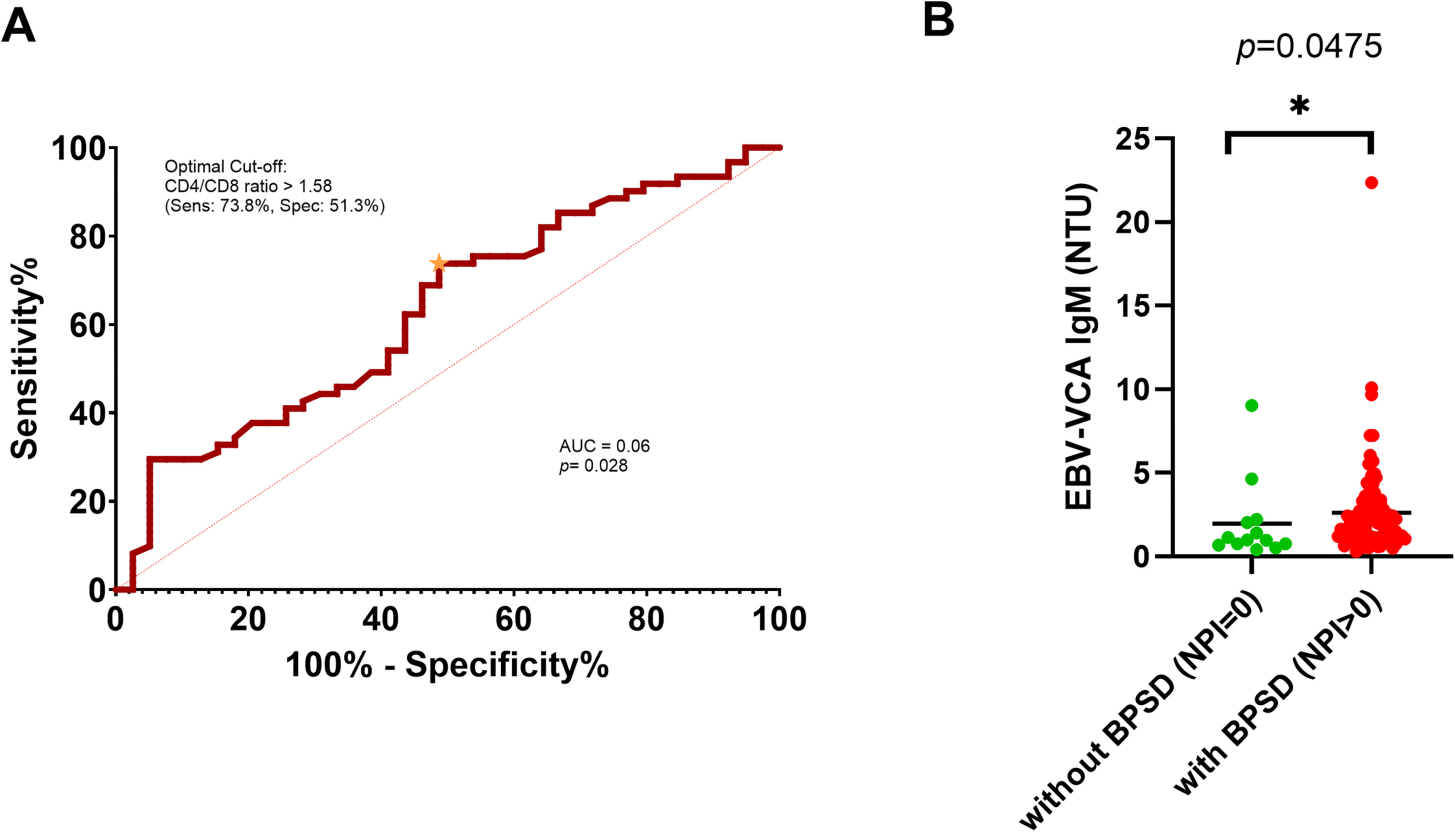
Peripheral immunological and virological biomarkers associated with Alzheimer’s disease clinical staging and symptom occurrence. **(A)** Receiver operating characteristic (ROC) curve analysis of the peripheral CD4/CD8 ratio for distinguishing patients with Alzheimer’s disease (AD) from those with mild cognitive impairment (MCI). The optimal cut-off value of >1.58 yields a sensitivity of 73.8% and a specificity of 51.3%, with an area under the curve (AUC) of 0.63. The star indicates the optimal cut-off point maximizing sensitivity and specificity based on AUC. **(B)** Threshold-associated priming effect of serological viral activity on BPSD occurrence. Scatter plot comparing EBV-VCA IgM titers between asymptomatic patients (NPI = 0) and those presenting with neuropsychiatric symptoms (NPI > 0). Patients with manifested BPSD exhibited significantly higher EBV-VCA IgM titers compared to the asymptomatic group (*p* = 0.0475, Mann-Whitney U test). Horizontal lines represent median values. The asterisk (*) denotes statistical significance between groups (*p* < 0.05). AD, Alzheimer’s disease; MCI, mild cognitive impairment; EBV-VCA, Epstein-Barr virus viral capsid antigen; ROC, receiver operating characteristic; AUC, area under the curve; NPI, Neuropsychiatric Inventory.

A total of 103 participants with probable Alzheimer’s disease or mild cognitive impairment were included in this study. As detailed in Table 1, the AD group was significantly older and exhibited greater cognitive impairment and more severe overall behavioral disturbances compared to the MCI group (all *p* < 0.01). To investigate macroscopic immune senescence across disease stages, we evaluated peripheral lymphocyte subsets. The AD group demonstrated a significantly higher CD4/CD8 ratio (2.26 ± 1.12) compared to the MCI group (1.87 ± 1.24, *p* = 0.028). To evaluate the diagnostic utility of this systemic T-cell imbalance, ROC curve analysis was performed. The CD4/CD8 ratio effectively distinguished AD from MCI (AUC = 0.63). Utilizing Youden’s Index, we identified an optimal cut-off value of >1.58, yielding a sensitivity of 73.8% and a specificity of 51.3% (Figure 1A). These findings establish systemic T-cell imbalance as a foundational biomarker reflecting the trajectory of advanced clinical disease staging.

### 3.2 Elevated viral titers and specific immune reductions act as priming factors for BPSD occurrence

**FIGURE 2:**
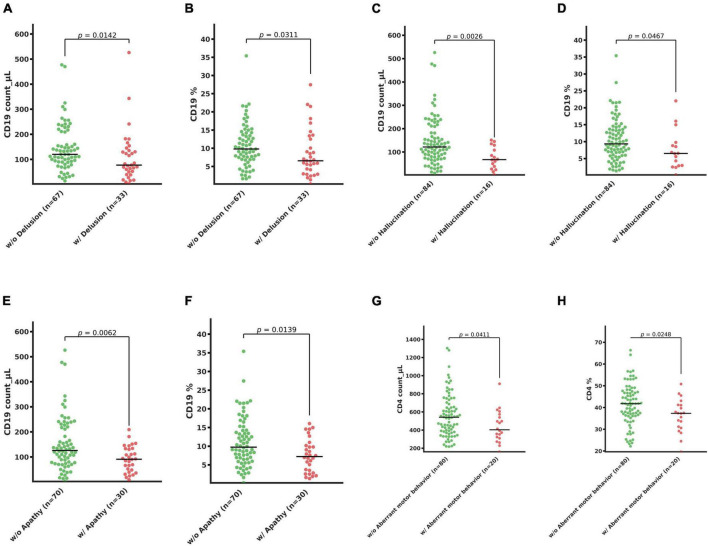
Peripheral immune cell profiles according to the presence of neuropsychiatric symptoms. Dot plots show comparisons of lymphocyte subsets within peripheral blood mononuclear cells (PBMCs) between participants with and without specific neuropsychiatric symptoms. **(A–F)** Absolute counts and percentages of CD19+ B cells in relation to delusions, hallucinations, and apathy. Both absolute counts and percentages were significantly higher in participants without these symptoms (*p* < 0.05, Mann–Whitney U test). **(G,H)** Absolute counts and percentages of CD4+ T cells in relation to aberrant motor behavior. Participants without aberrant motor behavior exhibited significantly higher absolute counts and percentages (*p* < 0.05). Horizontal lines represent median values.

To rigorously assess how immune and viral alterations correlate with BPSD without the statistical confounding of zero-inflation, we employed a two-step hurdle-like approach. Initial Mann-Whitney U tests were utilized to evaluate the threshold associated with symptom manifestation. We found that elevated EBV-VCA IgM titers were significantly associated with the presence of overall BPSD symptoms (NPI > 0 versus NPI = 0, *p* = 0.0475) (Figure 1B). Furthermore, examining specific symptom domains revealed that lower absolute counts and percentages of CD19+ B cells were significantly associated with an increased probability of manifesting delusions, hallucinations, and apathy (Figures 2A–F). Reduced CD4+ T-cell parameters were similarly linked to the presence of aberrant motor behavior (Figures 2G,H). These initial comparisons indicate that both serological viral activity and specific peripheral immune reductions operate as threshold-associated priming factors for the occurrence of neuropsychiatric phenotypes.

### 3.3 Reduction of adaptive immune cells continuously modulates global neurobehavioral severity

Subsequently, to evaluate whether these biological markers exhibit a continuous linear correlation with symptom severity, we performed Spearman rank correlations and simple linear regressions exclusively within the symptomatic cohort (NPI > 0). Interestingly, while elevated viral titers (EBV-VCA IgM and VZV-IgM) were associated with symptom occurrence, they lacked a continuous linear correlation with the severity of the Total NPI score.

**FIGURE 3:**
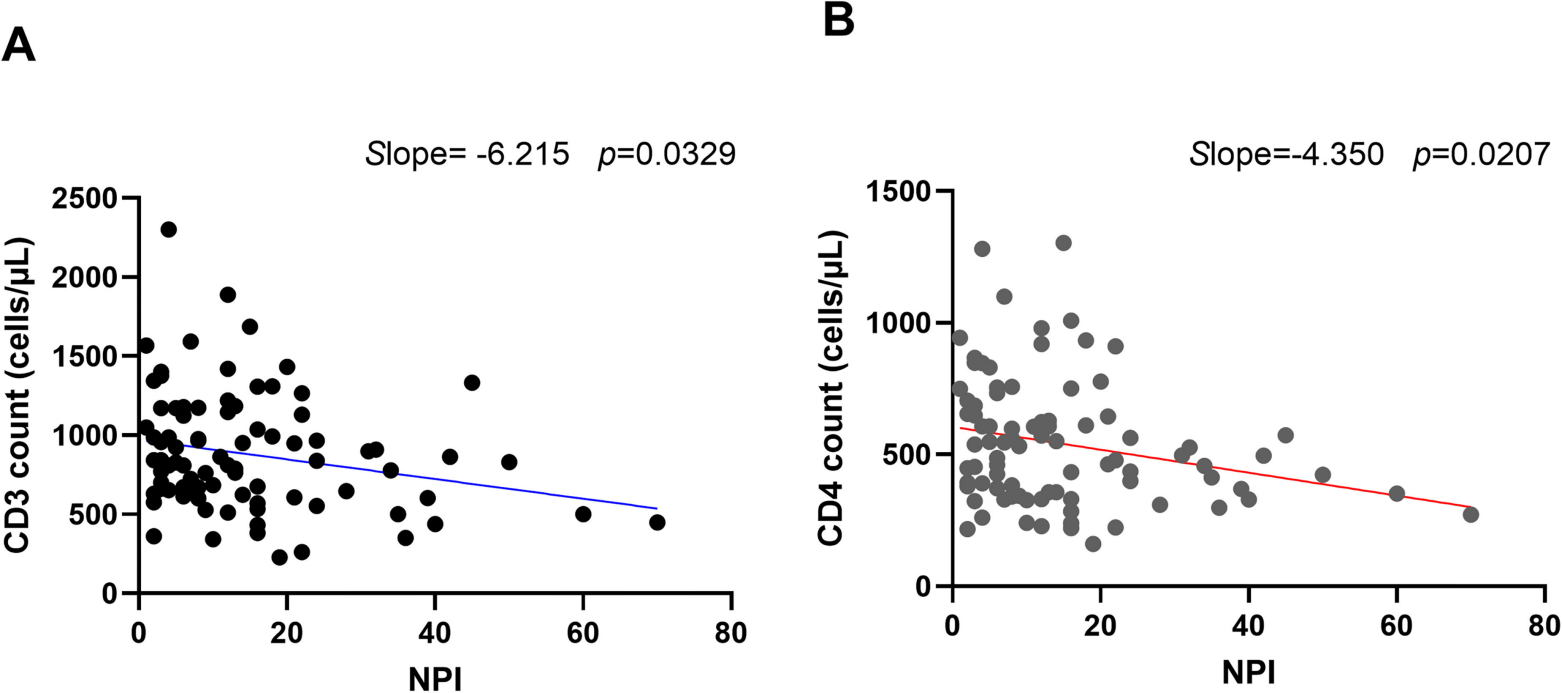
Dose-dependent modulation of continuous neurobehavioral severity by peripheral adaptive immune reserves. Scatter plots with simple linear regression lines demonstrating the continuous relationship between adaptive immune cell counts and global neuropsychiatric burden, analyzed exclusively within symptomatic patients (NPI > 0). **(A)** A significant negative correlation is observed between absolute CD3+ T-cell counts and Total NPI scores (Slope = −6.215, *p* = 0.0329). **(B)** Similarly, absolute CD4+ T-cell counts exhibit a significant inverse correlation with Total NPI scores (Slope = −4.350, *p* = 0.0207). These continuous inverse trajectories indicate that the reduction of the peripheral immune buffering capacity modulates the exacerbation of BPSD severity.

Instead, the continuous severity of the global neurobehavioral burden was robustly modulated by the reduction of adaptive immune reserves. Spearman rank analyses demonstrated a significant negative dose-dependent relationship between the Total NPI score and the absolute counts of CD3+ (*p* = 0.0373), CD4+ (*p* = 0.0171), and CD19+ cells (*p* = 0.0274). Simple linear regression further confirmed these inverse relationships, showing significant negative slopes for CD3 counts (slope = −6.215, *p* = 0.0329) (Figure 3A) and CD4 counts (slope = −4.350, *p* = 0.0207) (Figure 3B). Furthermore, after applying strict FDR correction (*Q* = 5%), the severity of nighttime behavior remained robustly and negatively correlated with the diminished pool of CD3 counts (*r* = −0.4064, *p* = 0.0023) and CD4 counts (*r* = −0.4939, *p* = 0.0001). This dynamic demonstrates that once the initial immunological threshold is crossed, the diminished pool of adaptive immune cells continuously modulates the worsening of neurobehavioral symptoms.

## 4 Discussion

To our knowledge, this study provides the first clinical evidence proposing an exploratory “dual-pathway framework” to elucidate the immunological and viral correlates underlying distinct BPSD in Alzheimer’s disease. While the cross-sectional nature of our dataset precludes the establishment of a mechanistically validated paradigm, our findings robustly support this conceptual framework. Rather than elucidating direct causal molecular mechanisms, this framework maps how macroscopic immunosenescence and elevated viral antibody titers clinically parallel distinct BPSD phenotypes. Rather than treating BPSD as a monolithic consequence of cognitive decline, our findings suggest that these highly heterogeneous symptoms correlate with the synergistic interplay between macroscopic immune senescence and elevated viral antibody titers.

### 4.1 The first pathway: T-cell senescence as a reflection of synergistic chronological aging and the immunological soil

The foundation of our framework is the systemic deterioration of the immune system. We identified that an elevated CD4/CD8 ratio (>1.58) showed a modest discriminative trend between clinical AD and MCI (Figure 1 and Table 1). In conventional infectious disease paradigms, a CD4/CD8 ratio inversion (<1.0) indicates immune exhaustion. However, in the context of aging and neurodegeneration, an elevated ratio often reflects a skewed T-cell pool characterized by the accumulation of senescent CD4+ T cells and a reduction of naïve CD8+ T cells, ultimately compromising peripheral immune regulation (Kaya et al., 2022; Weyand and Goronzy, 2016). This systemic T-cell imbalance serves as the immunological “soil,” marking the cross-sectional severity of clinical disease stages and rendering the brain increasingly vulnerable to inflammatory insults.

Importantly, we acknowledge that the elevated CD4/CD8 ratio observed in the AD group is deeply intertwined with chronological aging. As AD patients in our cohort were significantly older than the MCI group (Table 1), this ratio should be interpreted not merely as an independent, disease-specific predictor, but rather as a macroscopic index of “inflammaging.” In our exploratory dual-pathway framework, this age-related systemic immune exhaustion provides the necessary baseline environment, which subsequently allows specific biological factors (discussed in the second pathway) to correlate with neurobehavioral symptoms.

### 4.2 The second pathway: viral priming and the modulation of severity by adaptive immune reserves

Within this vulnerable immunological environment, specific biological factors prime the phenotypic presentation of BPSD. Our hurdle-like analysis revealed that elevated EBV-VCA IgM titers are significantly associated with the overall occurrence of BPSD symptoms. Rather than representing an acute systemic infection, these elevated IgM titers likely reflect a serological signature of ongoing immune interactions with latent viruses (Hatton et al., 2014; Zhang et al., 2022). This subclinical viral activity provides a baseline neuroinflammatory pressure that primes the central nervous system for behavioral disturbances. Concurrently, the initial reduction of specific immune subsets–such as CD19+ B cells, which are essential for immune tolerance (Kim et al., 2021; Plantone et al., 2022), and CD4+ T cells–is strongly associated with the manifestation of specific phenotypes, including psychosis, apathy, and aberrant motor behaviors (Figure 2).

A pivotal conceptual advancement of our dual-pathway framework, refined through addressing the zero-inflated data, is the functional bifurcation between symptom occurrence and continuous severity. Interestingly, while elevated viral titers are associated with the onset of symptoms, they do not exhibit a continuous dose-response correlation with global severity (Total NPI). This suggests that serological viral activity acts primarily as an initial immunological priming factor rather than a continuous driver of neurobehavioral deterioration.

Instead, our data demonstrate that the continuous severity of BPSD is robustly modulated by the reduction of adaptive immune reserves. We observed significant negative (inverse) correlations between the absolute counts of CD3+, CD4+, and CD19+ cells and the Total NPI score. This inverse relationship supports an “immune buffering” hypothesis: once the initial neuroinflammatory state is primed, the host’s ability to restrain the exacerbation of behavioral symptoms depends heavily on the remaining capacity of the peripheral adaptive immune pool. As these regulatory lymphocyte counts diminish, the peripheral immune system loses its capacity to exert anti-inflammatory control over the central nervous system (Mittli, 2023). This loss of regulation allows behavioral symptoms, such as nighttime behavior disruptions, to escalate in severity in a dose-dependent manner.

Therefore, our dual-pathway framework distinguishes two synergistic but functionally distinct phases: elevated viral antibody titers serve as the immunological priming factor associated with symptom occurrence, whereas the diminished capacity of adaptive immune reserves continuously dictates the magnitude of the clinical dementia crisis.

### 4.3 Study limitations and conclusions

Several limitations must be acknowledged. First, the cross-sectional design inherently limits our ability to establish definitive causality; the exploratory dual-pathway framework requires validation through longitudinal cohorts to map the precise temporal sequence of immune alterations and BPSD onset. Second, regarding the baseline age differences between the MCI and AD groups, we performed *post hoc* multivariate logistic regression analyses to assess the robustness of our findings against age-related confounding. Importantly, the significant associations between CD19+ B-cell reduction and specific BPSD domains (namely hallucinations and apathy) remained robust even after adjusting for age (adjusted *p* = 0.038 and *p* = 0.011, respectively). However, we acknowledge that the macroscopic CD4/CD8 ratio did not remain an independent predictor of AD staging when controlling for chronological age, largely due to the pronounced age discrepancy between the cohorts and the collinearity between immunosenescence and aging. Consequently, we emphasize that the CD4/CD8 ratio in this context predominantly serves as a marker of normal chronological immunosenescence that parallels cross-sectional clinical disease stages, rather than an independent, disease-specific diagnostic biomarker for longitudinal progression. Furthermore, given the highly skewed distribution of subclinical viral titers, the EBV associations were optimally captured via non-parametric univariate analyses. These nuances highlight the robust independent role of B-cell alterations in BPSD, while underscoring the need for age-matched longitudinal cohorts to untangle the precise variance contributed uniquely by neurodegeneration versus physiological aging. Third, the lack of central molecular confirmation (amyloid/tau) is a limitation, though our focus on clinically accessible peripheral biomarkers deliberately addresses translational real-world needs. Finally, because we assessed numerous immune subpopulations against 12 separate behavioral domains without stringent multiple comparison corrections for all initial steps, there is an inherent risk of Type I errors. Therefore, these findings should be interpreted as exploratory and hypothesis-generating, warranting validation in independent cohorts.

In conclusion, this study demonstrates that BPSD are deeply rooted in peripheral immune and viral dynamics. By bridging the bench-to-bedside gap using clinically accessible blood biomarkers, we propose an exploratory dual-pathway framework: elevated viral antibody titers (EBV-VCA IgM) act as a priming factor associated with the occurrence of behavioral symptoms, while the reduction of peripheral adaptive immune reserves (CD3+, CD4+, and CD19+ counts) serves as a continuous modulator of global neurobehavioral severity. These insights shift the therapeutic paradigm beyond traditional cognitive-centric approaches, suggesting that strategies aimed at restoring peripheral immune buffering capacity and managing latent viral burdens may hold profound translational potential for mitigating BPSD in vulnerable dementia populations.

## Data Availability

The original contributions presented in this study are included in this article/supplementary material, further inquiries can be directed to the corresponding author.
